# Glioblastoma Adjacent to Radiosurgically Treated Arteriovenous Malformation: A Case Report and Review of the Literature

**DOI:** 10.7759/cureus.22097

**Published:** 2022-02-10

**Authors:** Kent R Richter, Reed A Richter, Christoph Griessenauer, Edward A Monaco

**Affiliations:** 1 Neurological Surgery, Geisinger Medical Center, Danville, USA; 2 Neurological Surgery, McGovern Medical School at University of Texas Health Science Center at Houston, Houston, USA; 3 Department of Neurosurgery, Paracelsus Medical University, Christian Doppler University Hospital, Salzburg, AUT

**Keywords:** neurosurgery, stereotactic surgery, radiosurgery, arteriovenous malformations, glioblastoma

## Abstract

Stereotactic radiosurgery (SRS) is a noninvasive therapy for patients suffering from both benign and malignant intracranial pathologies. While SRS allows for increased precision and efficacy, significant risks have been reported, such as radiation necrosis. Although traditional radiation therapies are associated with a well-understood risk of causing tumors or inducing malignancy, the risks associated with SRS are not well understood. Here, we present the case of a patient who underwent SRS post-Onyx embolization of a Spetzler-Martin grade 4 left parasagittal arteriovenous malformation. Four years later, the patient presented with a high-grade glioma adjacent to where the SRS was targeted. SRS has fundamentally altered the way we treat intracranial pathologies. While the risks for SRS-induced glioma appear to be extremely low, this case illustrates that they ought to be considered. Here, we discuss the details of our case and explore the currently available literature. Knowing these potential risks will further aid physicians and patients balance the associated benefits and risks.

## Introduction

Stereotactic radiosurgery (SRS) has transformed neurosurgery by introducing new and innovative treatment methods for patients suffering from benign (arteriovenous malformation [AVM], meningioma, glomus tumor, pituitary adenoma, vestibular schwannoma, trigeminal neuralgia, essential tremor) [1] and malignant (brain metastases, gliomas) intracranial pathologies. SRS is noninvasive and allows for increased precision of delivered therapy, decreasing the risks and possible complications associated with both open surgery and traditional radiation therapies. Additionally, the majority of procedures are performed in an outpatient setting, reducing hospital length of stay, associated costs, and recovery. However, radiation-based therapies are associated with important risks, including radiation necrosis, extracranial secondary cancers [2], neoplasms in children [3], intracranial malignancies [4,5], and malignant transformation of benign intracranial masses [6]. The risk of intracranial tumors or malignancies resulting from SRS is controversial; reported risks for radiation-induced tumorigenesis range from 0.0% to 2.6% at 15-year follow-up [1,7], and 0.9% for malignant transformation of benign tumors [1]. More specifically, SRS-induced glioma has been reported after SRS for various lesions, including AVMs [8-10], melanoma metastases [11], meningioma [12,13], and other pathologies, with a reported risk of 0.04% at 15-year follow-up [7]. Thus, though the risk for SRS-induced glioma is likely low, it remains a valid concern that should be considered when discussing treatment methods with patients, especially for benign pathologies and in patients with longer life expectancies. In this case report, we discuss a patient who underwent Gamma Knife SRS post-Onyx embolization of a Spetzler-Martin grade 4 left parasagittal AVM in 2016. Four years after treatment, the patient presented with a high-grade glioma immediately adjacent to the targeted region.

## Case presentation

**Figure 1:**
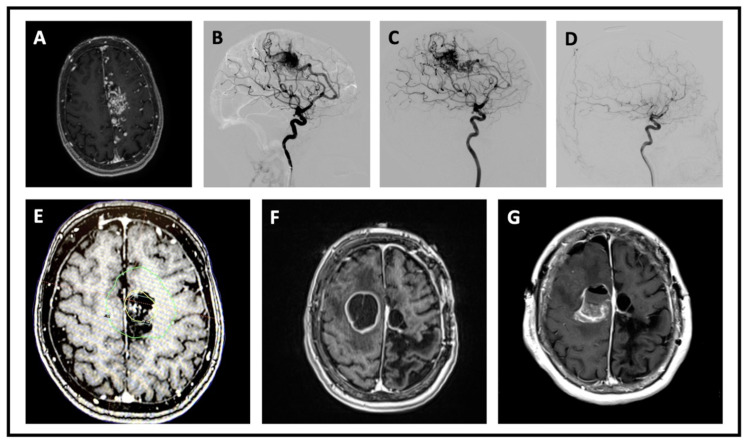
A: Axial T1 MRI with contrast demonstrating left-sided parasagittal AVM. B: Lateral digital subtraction angiography demonstrating AVM arising from the distal branches of the anterior cerebral artery. C: AVM status post-embolization. D: AVM status post-resection. E: Axial CT of the head with contrast demonstrating SRS treatment plan for left-sided parasagittal AVM; the yellow outline shows the target within the dose prescribed (23 Gy at the 50% isodose), the green line represents the 12 Gy line. F: Axial T1-weighted MRI with contrast demonstrating right-sided parasagittal glioma adjacent to the previously radiated and resected AVM on the contralateral side. G: Axial T1-weighted MRI with contrast demonstrating right-sided parasagittal glioma status post-resection. AVM: arteriovenous malformation; CT: computed tomography; MRI: magnetic resonance imaging

We present the case of a 63-year-old female with a medical history significant for a grade 4 left parasagittal AVM status post-Onyx embolization and SRS treatment (one session, prescription dose of 23 Gy at the 50% isodose), with subsequent rupture and surgical resection three years later causing residual right-sided hemiplegia. Two years after resection, the patient presented to the emergency room due to declining mental and functional status over a three-week period. The patient underwent magnetic resonance imaging that demonstrated a cystic and enhancing posterior right frontal lesion measuring 37 × 33 × 28 mm (Figure 1). On examination, the pertinent positive neurological findings included right upper extremity stiffness without movement to command or painful stimulation, left upper extremity stiffness with weak voluntary movements (3/5), and bilateral lower extremity weakness (2/5). Clonus was present in the left ankle. The patient’s cranial nerves were grossly intact, and light touch was intact bilaterally in the upper and lower extremities.

The patient underwent a bicoronal incision for right frontal parasagittal craniotomy and tumor resection with no intraoperative complications. Tumor tissue analysis was consistent with a giant cell glioblastoma, wild-type isocitrate dehydrogenase 1, p53 positive, with *methylguanine methyltransferase* gene promoter methylation. After the initial round of radiation therapy, the patient elected to pursue hospice therapy.

## Discussion

There is controversy surrounding the risk of radiation-induced gliomas and SRS. One measure that is used to classify radiation-induced tumorigenesis is the Cahan criteria. According to the Cahan criteria, for a tumor to be classified as being induced from therapeutic radiation, it must occur within the original radiation field but should not have been present on imaging at the time of initial irradiation; there must be a period between radiation exposure and the development of the second tumor; the second tumor must be histologically unique from the original target; and the patient cannot have a genetic syndrome that predisposes them to cancers [14]. Our patient met all these criteria. Though the reported risks for radiation-induced tumorigenesis are low (0.0-2.6% at 15-year follow-up [1,7], several cases of radiation-induced glioma after SRS have been reported [8-10]. Studies have demonstrated that the highest incidence of radiation-associated tumors occurs at the field peripheries where the dose is less than that at the field center [10,15]. There is no consensus regarding the dosage most likely to cause radiation-induced gliomas; however, some studies have suggested that lower-dose radiation delivered peripherally appears to increase the risk [10]. Our patient underwent SRS for her AVM with a prescription dose of 23 Gy at the 50% isodose in 2016. The glioblastoma that she presented with in 2021 was located within the peripheral radiation zone where the delivered dose was lower than the therapeutic dose delivered to the AVM (Figure 1).

Another point to consider is the system used to deliver SRS therapy, i.e., Gamma Knife versus linear accelerators. These systems are generally accepted to have comparable results in terms of coverage; however, Gamma Knife plans often have significantly steeper radiation fall off compared to CyberKnife and Novalis [16]. This may yield superior sparing of critical structures (brainstem, temporal lobe, cranial nerves) and decreasing the amount of radiation peripheral tissues receive, potentially reducing the risk of radiation-induced gliomas [17].

Much discussion also occurs surrounding the risks of radiation exposure associated with radiologic diagnostic and therapeutic planning, including computed tomography imaging, diagnostic angiograms, X-rays, etc. These are known risk factors for tumorigenesis, especially in younger patients [15,18]. There have been cases demonstrating radiation from computed tomography angiography and angiography might contribute to the risk of tumorigenesis, but not specifically glioma [18,19]. However, patients with AVMs have increased radiation exposure due to diagnostic angiograms and intraoperative fluoroscopy (i.e., Onyx embolization). These radiation doses accumulate and can possibly increase the risk of SRS-induced gliomas. Xhumari et al. explained that five out of the nine (55%) reported cases of SRS-induced gliomas were AVMs treated with SRS therapy [10].

## Conclusions

SRS is an important tool for the treatment of multiple intracranial pathologies. The patient presented here developed a glioblastoma in the brain tissue in immediate proximity to the SRS treatment volume for her AVM. Her case is consistent with the possibility of radiation-induced malignancy. The correlation between SRS and radiation-induced malignancies remains controversial, and because of its nature, it remains a challenging topic to investigate. Further studies are needed to better quantify the risk of SRS-induced gliomas. In particular, patients with benign diseases, longer life expectancies, and the need for numerous diagnostic and therapeutic procedures requiring radiation (i.e., angiograms, CT scans) may be at a higher risk of tumorigenesis after SRS.
